# Arenavirus infection correlates with lower survival of its natural rodent host in a long-term capture-mark-recapture study

**DOI:** 10.1186/s13071-018-2674-2

**Published:** 2018-02-08

**Authors:** Joachim Mariën, Vincent Sluydts, Benny Borremans, Sophie Gryseels, Bram Vanden Broecke, Christopher A. Sabuni, Abdul A. S. Katakweba, Loth S. Mulungu, Stephan Günther, Joëlle Goüy de Bellocq, Apia W. Massawe, Herwig Leirs

**Affiliations:** 1 Evolutionary Ecology Group, University of Antwerp, Antwerp, Belgium; 2 Bernhard-Nocht-Institute for Tropical Medicine, Hamburg, Germany; 3 Pest Management Center, Sokoine University of Agriculture, Morogoro, Tanzania; 4 Institute of Vertebrate Biology, Research Facility Studenec, The Czech Academy of Sciences, Brno, Czech Republic; 5 Department of Ecology and Evolutionary Biology, University of California, Los Angeles, USA; 6 Department of Ecology and Evolutionary Biology, University of Arizona, Tucson, USA; 7 Interuniversity Institute for Biostatistics and statistical Bioinformatics (I-BIOSTAT), Hasselt University, Hasselt, Belgium

**Keywords:** Arenavirus, Morogoro virus, Survival analysis, Capture-mark-recapture, Host-parasite interaction

## Abstract

**Background:**

Parasite evolution is hypothesized to select for levels of parasite virulence that maximise transmission success. When host population densities fluctuate, low levels of virulence with limited impact on the host are expected, as this should increase the likelihood of surviving periods of low host density. We examined the effects of Morogoro arenavirus on the survival and recapture probability of multimammate mice (*Mastomys natalensis*) using a seven-year capture-mark-recapture time series. *Mastomys natalensis* is the natural host of Morogoro virus and is known for its strong seasonal density fluctuations.

**Results:**

Antibody presence was negatively correlated with survival probability (effect size: 5–8% per month depending on season) but positively with recapture probability (effect size: 8%).

**Conclusions:**

The small negative correlation between host survival probability and antibody presence suggests that either the virus has a negative effect on host condition, or that hosts with lower survival probability are more likely to obtain Morogoro virus infection, for example due to particular behavioural or immunological traits. The latter hypothesis is supported by the positive correlation between antibody status and recapture probability which suggests that risky behaviour might increase the probability of becoming infected.

**Electronic supplementary material:**

The online version of this article (10.1186/s13071-018-2674-2) contains supplementary material, which is available to authorized users.

## Background

Classical microparasite evolution theory predicts a trade-off between virulence and transmission [1–3]. This trade-off balances virulence and within-host reproduction so that transmission is maximized over the lifetime of infection. Microparasites expressing intermediate levels of virulence are favoured under those conditions, as seen in several empirical examples of viruses with high transmission success (e.g. infections of myxoma virus in rabbits, HIV in humans, and cauliflower mosaic virus in *Brassica rapa*) [4–6]. However, not all studies found evidence for evolution towards intermediate virulence, but instead suggested evolution towards high or low virulence [7, 8].

Host population density is a key factor in determining whether low or high virulence will be optimal [9–11]. This mechanism can be understood in the framework of a trade-off between a microparasite’s competitive ability and its persistence. When transmission rates are lower at low host densities, a strain that can maintain a long infectious period (and thus low virulence) will have a persistence advantage over high virulence strains that kill the host or reduce its contact rate before transmission can happen. In contrast, a strain with a short infectious period but high reproductive rate (high virulence) will have an advantage at high host density, as it will outcompete strains with lower reproductive rate. These host density effects could be especially important during virulence evolution of wildlife parasites, as their hosts are more likely to experience strong density fluctuations [12].

For many wildlife microparasites, however, virulence levels have not been assessed, and conflicting results have been found regarding the virulence of arenaviruses (Arenaviridae, *Mammarenavirus*) in their natural rodent host [13]. Most arenaviruses seem to be restricted to a single rodent species or even sub-species [14, 15] suggesting adaptation to those specific hosts. Some arenaviruses can also infect humans, potentially causing severe disease or death. Lassa arenavirus (LASV) for example can cause Lassa fever which annually affects around 200,000 people in West Africa [16, 17]. Other examples include Junín, Machupo, Guanarito and Sabia arenaviruses that cause sporadic outbreaks of haemorrhagic fevers in South America. Although the pathogenic effects of arenaviruses on humans are relatively well-documented, little is known about their effects on their natural rodent hosts [18, 19].

Most information about arenavirus virulence in rodents is derived from laboratory inoculation studies, e.g. lymphocytic choriomeningitis virus (LCMV) in *Mus musculus*, LASV and Morogoro virus (MORV) in *Mastomys natalensis*, Machupo virus in *Callomys callosus* and Junin virus in *Callomys musculinus* [20–23]. The inoculated rodents from these studies typically remained symptom-free despite temporary high viral loads, although severe disease symptoms have also been observed for LCMV [24–26]. Still, several factors that influence virulence can differ between laboratory and natural settings, including variation in infection route or dose, viral strain, stress levels or individual life histories [27]. In order to examine the effects of arenavirus infection in natural conditions, we recently analysed capture-removal studies in which we related body condition variables (head-body length, body weight, fecundity and maturation rate) of wild *M. natalensis* to infection status [13]. Although we found no adverse relationship between MORV infection and body condition, we were not able to exclude the possibility that animals become lethargic or die quicker due to infection and thus have a lower capture probability. For this reason we now investigate whether MORV reduces the survival and recapture probability of *M. natalensis* (its reservoir host) using a seven-year capture-mark-recapture (CMR) dataset.

MORV infection in *M. natalensis* provides an interesting model system for examining parasite-host interactions, as the ecology and evolution of both the virus and the rodent host have been studied intensively [15, 23, 28–31]. In particular, it provides a safe alternative to studying closely related but pathogenic arenaviruses such as LASV. MORV is endemic to East Africa where seroprevalence in *M. natalensis* has been found to range between 5 and 50% [30, 32]. In this region, *M. natalensis* populations experience seasonal density fluctuations, generally ranging from 20 to 250 individuals per hectare [33]. These fluctuations are the result of seasonal breeding, driven by a bimodal rain pattern with short (November–December) and long (March–May) rainy periods [34]. Reproduction starts shortly after the long rains and continues until the end of the dry season in October. Population density peaks around November after which it decreases rapidly, probably due to a combination of competition for resources due to high population density and decreasing food availability, and survival effects of intense rainfall [34]. Despite the seasonal periods of low densities, MORV manages to persist and can be detected even at very low host densities [30]. This may be surprising for a (mainly) directly transmitted parasite of which the host’s contact rate is assumed to be density-dependent and infection predominantly acute, and is probably only possible if a proportion of animals becomes infected chronically [23, 27, 30, 35].

We hypothesize that MORV virulence is low in its reservoir host *M. natalensis*, as longer host survival combined with chronic infection in some animals would allow MORV to persist during the seasonal periods of low host population density [36]. For this reason, we predict that no adverse relationship exists between MORV infection status and *M. natalensis* survival probability.

## Methods

### Study area and trapping

A capture-mark-recapture study was performed between May 2010 and April 2017 on a mosaic field (maize and fallow land) on the campus of the Sokoine University of Agriculture in Morogoro, Tanzania (6°51′S, 37°38′E). A robust trapping design was used with trapping sessions conducted every month (primary capture occasion) for three consecutive nights (secondary capture occasions). Sherman live traps (Sherman Live Trap Co., Tallahassee, FL, USA) were placed in a rectangular 300 × 100 m grid and spaced evenly at 10 m intervals. The traps were baited in the evening with a mixture of peanut butter and corn flour and checked in the morning. Trapped animals were transported to the lab, where species, sex, weight and reproductive status were recorded [28, 29]. Mice were considered to be adults if signs of sexual activity were observed (scrotal testes in males; perforated vagina, lactating nipples or pregnancy in females). Blood samples were taken from the retro-orbital sinus and preserved on prepunched filter paper (± 15 μl/punch; Serobuvard, LDA 22, Zoopole, France). Blood was only sampled once per monthly session, so if an animal was recaptured in the same three-day session, blood was not taken again. Each rodent was individually marked by toe clipping [37], and released at the location where it was trapped.

### Serology

Filter papers were dried and stored in the dark at ambient temperature in a locked plastic bag with dehydrating silica gel. Since 2014, blood samples were preserved at -20 °C after drying. Dried blood spots on filter paper were punched out and eluted in a 100 μl solution of phosphate buffer saline and 0.25% NH_3_ [38]. Blood samples were analysed for the presence of antibodies (Ab) by indirect immunofluorescence assay using MORV-infected Vero cells as antigens and polyclonal rabbit anti-mouse IgG (Dako, Glostrup, Denmark) as secondary antibodies [31].

### CMR data

The CMR dataset consisted of 8274 separate captures of 3884 unique *M. natalensis*, of which 855 individuals were seropositive at least once, and 168 seroconversion events were detected where the infection status of animals changed from Ab-negative to positive in between trapping sessions. Antibody status was used as an indication of recent or past MORV infection, except for very young individuals that still might have maternally-derived Ab [39]. We therefore removed the youngest animals (body weight at first capture < 15 g) from the dataset. A small number of animals (*n* = 43) showed an apparent loss of Ab. These negative samples were considered to be false negatives due to Ab titers dropping below the detection threshold of the Ab-assay, as *M. natalensis* normally exhibits long-term Ab production after MORV infection [23, 27].

The CMR data were analysed using R [40] package ‘marked’ [41], which provides functions that allow efficient interfacing with CMR analysis software MARK [42]. For survival analysis, we assumed a multivariate multistate Cormack-Jolly-Seber model that allows for parameter estimations in systems where different states (e.g. Ab-positive/negative) can be assigned to surviving individuals [43]. All parameter estimates were based on the primary capture occasions (i.e. the monthly trapping occasions), which were standardized to a time span of 30 days. Because time intervals between primary occasions varied between 22 and 55 days in reality, we included these differences into the model’s design matrix.

### Goodness of fit test

A goodness of fit (GOF) test was carried out with the program U-CARE to evaluate possible effects of confounding factors [44, 45]. Major deviations against assumptions on ‘transience’ and ‘trap-dependence’ were found (see Results). The null hypothesis on ‘transience’ states that there is no difference in the re-encounter probability of newly trapped and recaptured individuals. Because we were interested in survival of resident animals only (not in migration), we decided to remove all transient animals from the CMR data set [29]. Transient animals were defined as individuals that were captured only once during one secondary capture occasion. These individuals were (most likely) not re-encountered because they moved outside of the trapping grid, and not because they died shortly after release. Removing transient individuals obviously solved the problem against the assumption on ‘transience’, but was only possible on the condition that Ab-prevalence did not differ between transient and resident individuals. This assumption was tested using a generalized linear model with binomial distribution and logit-link function (see Results).

The null hypothesis on ‘trap-dependence’ states that when individuals are caught, they become aware of the trap and will actively seek or avoid it at the next trapping occasion (e.g. *M. natalensis* becomes trap-happy in our dataset). This effect is likely to be strongest just after a capture occasion. In order to correct for trap-awareness, we implemented an immediate trap effect in the model using trappability states, in which individuals were able to move in a Markovian way between a ‘trap-aware’ state (after occasions when they are captured) and a ‘trap-unaware’ state (after occasions when they are not captured) [46].

### Modelling

The multistate model estimates three probabilistic events: the monthly probability that animals survive (Φ), the monthly probability that animals are recaptured (P) given that they were still alive, and the monthly probability that animals move between states (transition, ψ) given that they were alive in that state [43]. Trapped individuals were assigned an infection and a trappability state on each capture occasion: (i) Ab-negative and trap-aware; (ii) Ab-positive and trap-aware; (iii) Ab-negative and trap-unaware; (iv) Ab-positive and trap-unaware; and (v) not captured. Because *M. natalensis* is assumed to stay MORV Ab-positive during its entire life, transitions from Ab-positive to Ab-negative states were not allowed in the model [23].

Each of the parameters (Φ, P, ψ) was fitted by the following fixed factors: time, age, and infection status. It was not possible to fit fully time-dependent models, because our CMR study contained too many capture occasions (84) which would overparameterise the models. We therefore simplified the fully time-dependent model into a seasonal one (breeding season: May-October; non-breeding season: November-April), as seasonal effects have been shown to account for the largest variation in survival of *M. natalensis* in Morogoro [29, 47]. We did not include a year effect in the models for several reasons that are further explained in Additional file 1: Figure S1. An age factor was included into the models to correct for the positive relation between *M. natalensis*’ age and Ab-prevalence [30]. Older animals are more likely to be Ab-positive because Abs remain present after infection throughout an animal’s lifetime, and older animals have had more opportunities to have encountered the infection than younger animals. Without this correction, we might have found that infected individuals have lower survival only because they are old. We used the logarithm of body weight on first capture as proxy for age, as we recently found that body weight is not affected by MORV infection and relates linearly to log(eye lens weight), which is an unbiased indicator of age in rodents [13, 28]. However, as it is known that variation in body weight increases significantly in adult animals, we removed all individuals of which the body weight was higher than 35 g on first capture [28].

The most complex model contained all possible main effects and their interactions. A trappability factor was added to the recapture and transition models, but not to the survival models. The modelling itself occurred in subsequent steps: first we modelled transition, then recapture and finally survival. The models were ranked according to Akaike information criterion (AICc) and the one with the lowest AICc was selected as starting point for the next modelling step. During the first steps, survival and/or recapture were fixed and modelled by an interaction between season and weight and a trappability effect (for recapture only).

All models were implemented in R using the R packages *marked*, *mvtnorm*, *dplyr* and *ggplot2* [41, 48, 49]. The R code can be found in Additional file 2 of the supplementary material. After removal of the transient individuals and individuals with a body weight < 15 g and > 35 g, the remaining data set contained 1219 individuals of which 325 were at least once seropositive and 118 seroconverted.

## Results

### Goodness of fit test

The GOF test showed major deviations against assumptions on transients (TEST 3.SR one sided test for transience, *χ*^2^ = 83, *df* = 63, *P* = 0.005) and trap-dependence (TEST M.ITEC, *χ*^2^ = 132 *df* = 44, *P* < 0.001, animals became trap-happy). Because Ab-prevalence was not significantly different between transient (individuals that were captured only once during one secondary trap interval) and resident animals [GLM, *χ*^2^ = 1.6, *df* = 1, *P* = 0.201, Ab prevalences were 20% (95% CI: 18–21%) and 18% (95% CI: 16–20%), respectively], we could safely remove the transient animals from our data set. More than half of the animals (56%) were captured only once in the three hectare open grid. This pattern matched previous findings and suggests that the recapture probability at the primary trapping session is fairly low for *M. natalensis* in this experimental setup [29]. While the deviation on transients hereafter disappeared (TEST 3.SR one sided test for transience, *χ*^2^ < 1, *df* = 59, *P* > 0.999), the deviation on trap-dependence remained in the reduced data set (TEST M.ITEC: *χ*^2^ = 170, *df* = 57, *P* < 0.001). We corrected for this trap-dependence by implementing two possible trappability states in the models (see Methods).

### Model selection

The transition model with the lowest AICc value included two interactions: one between infection (Ab-presence) and season and one between season and weight (ψ_I*S + S*W_, AICc = 8660, -2lnL = 8628, par = 16) (Table 1). This model was 1 AIC unit removed from a transition model that fitted second best and which also contained interactions between infection, weight and season (ψ_I*W*S,_ AICc = 8661, -2lnL = 8625, par = 18). After modelling transition we modelled recapture. The recapture model with the lowest AICc included an infection effect only (P_I_, AICc = 8655, -2lnL = 8627, par = 14). Two other recapture models were only 1 AICc unit removed from the best fitting recapture model. These models contained an additive effect between infection and season (P_I + S_, AIC = 8656,-2lnL = 8626, par = 15) or an interaction between infection and season (P_I*S_, AIC = 8656, -2lnL = 8624, par = 16). During the modelling of survival, we found two models that had the same lowest AICc value. The first model included an added effect between infection and season (Φ_I + S_, AICc = 8645, -2lnL = 8619, par = 13). The second model included an interaction between infection and season (Φ_I*S_, AICc = 8645, -2lnL = 8617, par = 14). One survival model was 1 AICc unit removed from the two best fitting models. It contained an additive effect between infection, season and weight (Φ_I + S + W_, AICc = 8646, -2lnL = 8618, par = 14). We eventually choose the survival model with the lowest AICc value and lowest number of parameters. This final model contained the following factors: ψ_I*S + S*W_, P_I_, Φ_I + S_.

**Table 1:** Modelling of transition, recapture and survival. Highlighted (bold) models were selected in each step and used as starting point for the subsequent step

Transition	Recapture	Survival	AICc	Lnl	Par
I*S + S*W	S*W	S*W	**8660**	**8628**	**16**
I*W*S	S*W	S*W	8661	8625	18
I + W*S	S*W	S*W	8662	8632	15
I*S + W	S*W	S*W	8662	8632	15
I*W + S*W	S*W	S*W	8662	8630	16
I*W + I*S + S*W	S*W	S*W	8663	8629	17
I + W + S	S*W	S*W	8664	8636	14
I*W	S*W	S*W	8664	8636	14
I + W	S*W	S*W	8665	8639	13
I*W + S	S*W	S*W	8668	8638	15
I*S + I*W	S*W	S*W	8668	8636	16
I*S + S*W	**I**	S*W	**8655**	**8627**	**14**
I*S + S*W	I + S	S*W	8656	8626	15
I*S + S*W	I*S	S*W	8656	8624	16
I*S + S*W	I*S + W	S*W	8657	8621	17
I*S + S*W	~	S*W	8657	8631	13
I*S + S*W	I + W	S*W	8657	8627	15
I*S + S*W	I + S + W	S*W	8658	8626	16
I*S + S*W	S	S*W	8659	8631	14
I*S + S*W	W	S*W	8659	8631	14
I*S + S*W	I*W	S*W	8659	8627	16
I*S + S*W	I*S + W*S	S*W	8659	8623	18
I*S + S*W	I*S + W*I	S*W	8659	8623	18
I*S + S*W	S + W	S*W	8660	8630	15
I*S + S*W	I*W + S	S*W	8660	8626	17
I*S + S*W	S*W + I	S*W	8660	8626	17
I*S + S*W	I*S + S*W + I*W	S*W	8661	8623	19
I*S + S*W	S*W	S*W	8662	8630	16
I*S + S*W	I*W + W*S	S*W	8662	8626	18
I*S + S*W	I*S*W	S*W	8662	8622	20
I*S + S*W	**I**	**I + S**	**8645**	**8619**	**13**
I*S + S*W	I	I*S	8645	8617	14
I*S + S*W	I	I + S + W	8646	8618	14
I*S + S*W	I	I*S + W	8647	8617	15
I*S + S*W	I	I*W + S	8647	8617	15
I*S + S*W	I	I*S + W*I	8647	8615	16
I*S + S*W	I	S*W + I	8648	8618	15
I*S + S*W	I	I*S + W*S	8649	8617	16
I*S + S*W	I	I*W + W*S	8649	8617	16
I*S + S*W	I	I*S + S*W + I*W	8649	8615	17
I*S + S*W	I	I*S*W	8650	8614	18
I*S + S*W	I	S	8652	8628	12
I*S + S*W	I	S + W	8653	8627	13
I*S + S*W	I	S*W	8655	8627	14
I*S + S*W	I	I	8846	8822	12
I*S + S*W	I	I + W	8846	8820	13
I*S + S*W	I	I*W	8848	8820	14
I*S + S*W	I	~	8863	8841	11
I*S + S*W	I	W	8863	8839	12

*Abbreviations*: I, infection (antibody positive or negative); S, season (breeding and non-breeding season); W, weight (proxy for age of *Mastomys natalensis*); AICc, sample size corrected version of Akaike information criterion; -2Lnl, -2*log likelihood; Par, number of identifiable parameters; ~, model with no fixed effects

### Survival estimates

**Fig. 1:**
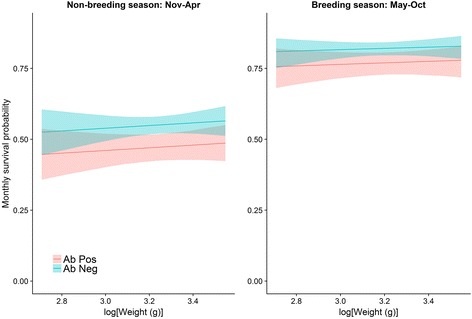
Monthly survival probability of MORV Ab-negative (blue) and Ab-positive (red) *M. natalensis* in function of log(body weight), a proxy for rodent age. The survival probability is given for the non-breeding (left) and breeding season (right). Solid lines and envelopes represent probabilities that an animal survived and 95% confidence interval (CI)

The best fitting survival model included differences between season and infection status (Fig. 1). During the breeding season, Ab-positive individuals had a monthly survival probability of 0.77 (95% CI: 0.72–0.80) compared to 0.82 (95% CI: 0.80–0.84) for Ab-negative animals. During the non-breeding season, Ab-positive individuals had a monthly survival probability of 0.47 (95% CI: 0.43–0.52) compared to a survival probability of 0.55 (95% CI: 0.52–0.58) for Ab-negative animals.

### Recapture estimates

The best-fitting recapture model included an infection effect only (P_I_). The recapture probability of Ab-positive animals was higher than the recapture probability of Ab-negative animals irrespective of age or season [estimate: 0.08 (95% CI: 0.11–0.03)]. After correction for trap-dependency effects, the recapture probability of Ab-positive animals was 0.31 (95% CI: 0.25–0.37) compared to 0.23 (95% CI: 0.20–0.25) for Ab-negative animals.

## Discussion

The survival models indicate that the presence of anti-MORV Ab correlates with a 5–8% lower survival probability of *M. natalensis* in natural conditions. In our previous study we did not observe any adverse effects of MORV on the hosts’ body condition [13], but these two results are not necessarily contradictory. Parasites can impair host health through a variety of mechanisms that may affect survival probability but not the body condition parameters that we evaluated (body weight, body length, and reproductive maturity), such as behavioural changes or an increased susceptibility to secondary infection. Furthermore, the effect on survival probability observed here was small, so the power to detect very small effects on body condition parameters in our previous study, with more limited sample size (*n* = 743; 73 were Ab positive), was perhaps not high enough.

This field study and the previous one [13] are in line with a laboratory inoculation experiment that showed how body weight of inoculated *M. natalensis* can decrease between days 7 and 15 post-infection (approx. 7% of normal body weight in 40% of inoculated animals) but recovers quickly without affecting further growth rates [23]. While such rapid recovery might explain why we did not observe any significant effects during the previous study, the temporary decrease in body weight does suggest that MORV can induce adverse effects in some individuals. Because severity of disease is likely higher in stressful natural than in stress-free laboratory conditions, a temporary disease effect could indeed explain the small negative effect on survival probability. Note also that the use of Ab as indicator of recent or past infection could have induced some bias in this study. Ab-positive individuals might experience only small adverse effects because of their effective immune response, while infected immunocompromised individuals (categorized as Ab-negative in our dataset) might not recover and die quickly. If immunocompromised individuals indeed exist in the wild [27], including both Ab and MORV RNA data might have resulted in bigger differences in survival probability.

Host fitness and infection status have also been associated for several other rodent-borne parasites. Hantavirus infections were initially assumed to be asymptomatic in rodents as no obvious pathology (such as reduction in body weight or fecundity) had been observed [50–52]. However, recent CMR studies showed that hantaviruses can affect the survival probability of rodents depending on sex and reproductive status. For example, Puumala hantavirus decreased survival of reproductively inactive bank voles (*Myodes glareous*) by 14%, while Sin Nombre hantavirus decreased survival of male deer mice (*Peromyscus maniculatus*) by 13% [53–55]. Cowpox virus infection in voles and mice was also initially assumed to be asymptomatic [56, 57], but CMR studies showed that infections can correlate both positively and negatively with survival probability depending on the season. A positive relation between cowpox infection and survival probability was for example observed in bank voles and wood mice (*Apodemus sylvaticus*) during summer (the reproductive season), while negative effects were observed during winter [58]. Infected field voles (*Microtus agrestis*) had an overall lower survival probability of 10–22% compared to uninfected field voles [58, 59].

**Fig. 2:**
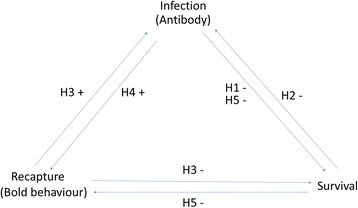
Schematic view of the possible relationship between infection, survival and recapture as described in the discussion. *Abbreviations*: ‘+’ sign, two variables are proportionally related; ‘-’ sign, two variables are inversely proportionally related; H, hypothesis

The observed negative relationship between survival and MORV Abs may also be explained by other not mutually exclusive hypotheses, termed here H1-H5 (Fig. 2). One possibility (H1) is that MORV has a direct negative effect on survival of *M. natalensis* because of the costs of clearing the virus (Fig. 2, H1). Another possibility (H2) is that Ab-positive mice have a lower survival probability because of prior poor condition (e.g. secondary infections) that might increase susceptibility to MORV (Fig. 2, H2). This situation has been observed for cowpox virus infections in *M. agrestis* [60]. Yet, this seems unlikely for MORV as no negative relationship exist between MORV infection and *M. natalensis*’ body condition [13]. Alternatively (H3), the negative association between MORV infection and host survival may be explained by confounding host behavioural traits that affect both the probability of survival and infection (Fig. 2, H3). For example, individuals with a risky lifestyle could be more susceptible to both predation and parasitism [61]. Vanden Broecke et al. [62] showed that such consistent behavioural differences exist in *M. natalensis*, and although they found no significant relation between explorative behaviour and MORV infection (possibly due to the low number of infected animals in their study), it is not unlikely that other personality types such as boldness do influence the probability of becoming infected. Since bolder individuals might be more likely to enter traps [63] and our results show a higher recapture probability (~ 8%) for Ab-positive individuals, it could indeed be possible that animals with a bolder personality are more at risk of becoming infected.

The positive relation between Ab-status and recapture probability can also be explained by two other hypotheses. Because it has been shown that arenaviruses can affect host behaviour directly (e.g. persistent LCMV infections impair the learning capacity of laboratory mice), MORV might increase the recapture probability of *M. natalensis* by decreasing neophobic behaviour in the rodent (Fig. 2, H4) [64–66]. Similarly, *Toxoplasma gondii* decreases neophobic behaviour in brown rats (*Rattus norvegicus*) which is suggested to increase the rat’s recapture probability and susceptibility to predators [67]. Otherwise (H5), the positive relation between infection and recapture probability might be explained indirectly by unavoidable side effects of infection on host health (i.e. sickness effects) (Fig. 2, H5) [61]. Infected *M. natalensis* might need to recover from infection and therefore increase risk-taking behaviour to search for extra food, which might result in a higher recapture probability. In other words, the observed behaviour may be a consequence of rather than a risk factor for infection.

## Conclusions

We found a significant negative relation between *M. natalensis* survival probability and MORV Ab-status. However, the effect of infection was small (5–8%) and probably negligible compared to the effects of environmental factors such as rainfall (25–30%), which is known to be an important driver of survival and reproduction in *M. natalensis* [29, 34]. Since we previously observed no relationship between MORV and the body condition or reproductive maturity of its host and now only a small effect on its survival [13], it seems that MORV does not significantly affect the population dynamics of *M. natalensis*. Combined, our two field studies suggest that MORV virulence in its natural rodent host is low, which could be the result of adaptation to persistence in the seasonally fluctuating host populations.

## Additional files

Additional file 1: Figure S1. Correlation between monthly survival probability and maximum antibody prevalence measured per year (2010 until 2017). (DOCX 84 kb)

Additional file 2: Supplementary R code of the models. (R 5 kb)

## Abbreviations

Ab: Antibody
AIC: Akaike information criterion
CI: Confidence interval
CMR: Capture-mark-recapture
LASV: Lassa virus
LCMV: Lymphocytic choriomeningitis virus
MORV: Morogoro virus
