# Supplementary material for: Arenavirus infection correlates with lower survival of its natural rodent host in a long-term capture-mark-recapture study
Source: Parasit Vectors. 2018 Feb 8;11:90. doi: 10.1186/s13071-018-2674-2 (PMC5806307; doi:10.1186/s13071-018-2674-2)
Supplement: Supplementary file 1 — Correlation between monthly survival probability and maximum antibody prevalence measured per year (2010 until 2017). (DOCX 84 kb) [file 13071_2018_2674_MOESM1_ESM.docx]

**Additional file 1**

**Supplementary information**

Explanation for not using ‘year’ as variable in the models

We did not include ‘year’ as variable in the models for four reasons:

- We were not primarily interested in the effect of year on survival but only in the effect of MORV infection. We assumed that it was only necessary to include season and weight as fixed factors in the models, as we observed significant differences in prevalence according to season (Mariën et al, unpublished data) and because it was essential to correct for an age effect (as explained in the main text). In an ideal scenario we would have implemented year as a random variable in the models, but there were only seven years, which is not enough for the implementation of a random variable.
- Because we also observed differences in prevalence between years, we tested if there was a correlation between yearly prevalence and yearly survival: we found a positive correlation (Figure S1). This positive correlation is most likely caused by the fact that animals which survive longer become older, and are therefore more likely to develop antibodies due to the longer time window they have been alive. Because we already corrected for age effects, we assumed that it was not necessary to correct for this positive correlation between prevalence and survival. If we would have found a negative correlation between yearly prevalence and yearly survival probability, it would have been necessary to include year as factor in the model. In that case, year would be a confounding factor which might have explained the negative correlation between MORV Ab presence and survival probability. For example, if survival would have been low during certain years (e.g. because of climate conditions) but Ab prevalence high, it would be logic to find a negative correlations between MORV infection and survival. This was however not the case.
- If we included year as fixed effect in the models, we would have increased the number of parameters substantially (number of parameters x7). This would have made the models too complex for the amount of data that we had. It would have been especially problematic in our situation, as we used the ‘AIC approach’ for model selection which is often claimed to over-fit models which are overly parametrized (relative to the true general model).
- Even if we added year as fixed effect to the models, we still selected a model that included an infection effect as the best model. Estimates of the infection effect were almost similar between models with and without year. The errors on the estimates were however considerably larger in models with year. This suggests that adding or leaving out year would not have severely affected our results


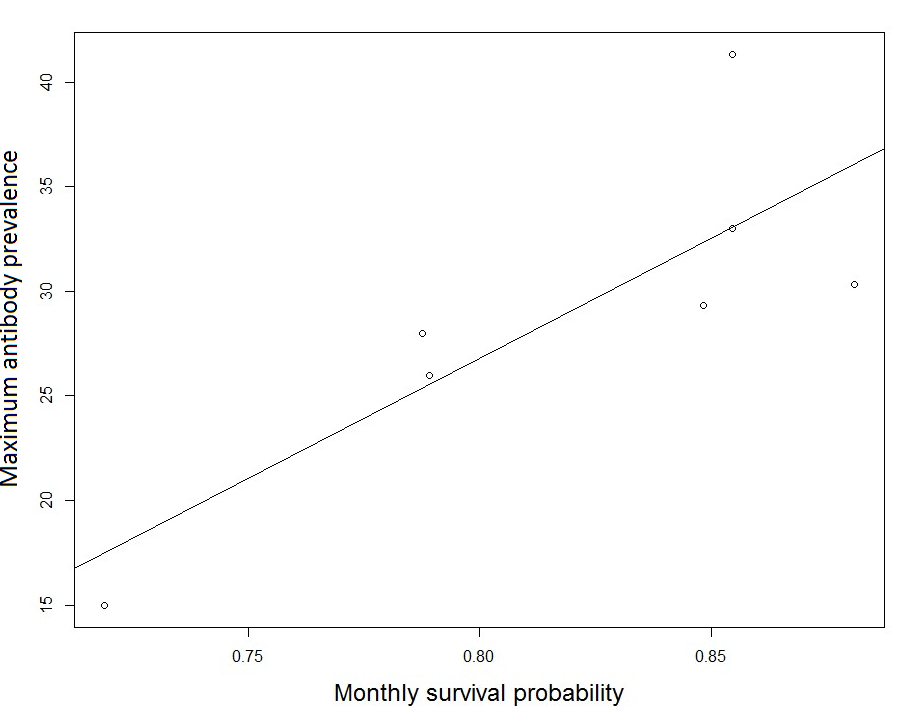


**Figure S1** Correlation between monthly survival probability and maximum antibody prevalence measured per year (2010 until 2017).
